# A Story of Hope: How a Community Project is Transforming the Lives of Filipino Children With Intellectual and Developmental Disabilities

**DOI:** 10.1177/2333794X231211215

**Published:** 2023-11-12

**Authors:** Lance Vincent C. Sese, Ma. Celina L. Guillermo

**Affiliations:** 1 Nuffield Department of Primary Care Health Sciences, University of Oxford, Oxford, UK; 2 Department for Continuing Education, University of Oxford, Oxford, UK; 3 Ateneo School of Medicine and Public Health, Pasig, Metro Manila, Philippines

**Keywords:** autism, intellectual disabilities, developmental disabilities, child health, Philippines

## Introduction

The issue of social inequality became more prevalent during the pandemic and with it, the call for preferential option toward the vulnerable.^1^ Not only do we protect them from sickness and death, but we are also called to improve their quality of life. Thus, it is imperative that in this call for inclusivity, we address the gap, one group at a time—for example, children with special needs. The project discussed in this perspective article exemplifies the importance of collaboration in bridging the gap to create a more holistic environment for Filipino children with special needs. This article then serves to highlight the importance of addressing social inequality and advocating for inclusivity, focusing on improving their quality of life and empowering their caretakers.

## International and Local Context

In March 2022, the WHO published an article highlighting the need to address the global burden of autism, with a particular focus on improving government efforts and promoting enabling environments.^2^ Despite multiple studies reporting different data on the prevalence of autism, it is estimated that, on average, 1 in a 100 children has autism worldwide.^3^ An archipelago consisting of more than 7000 islands, the Philippines has 2462 reported autism cases with Metro Manila and Region IV-A contributing 34% and 16% from August 2020 to October 2021 respectively.^4^ Although there is a gradual increase in mental disability awareness in many developing countries, people with autism are still often overlooked and excluded. With no recent data available, the disparity in reported cases within the provincial regions in the Philippines can potentially reveal the need for early and proper assessment and subsequent intervention. Possible underreporting of cases within regions with high poverty incidence can be attributed to a lack of available specialists for proper assessment. Consequently, immediate intervention and holistic educational support, which can drastically improve the child’s quality of life, can be provided once the proper assessment has been done.^2^ Furthermore, at the height of the lockdown last 2020, another challenge loomed as there was a shift to distance learning. The teachers acknowledged that children with special needs required individualized and tailor-fit programs which might be more difficult to deliver in an online set-up and thus, the need for the local and national government to be more involved.^5^

Echoing the drive for Universal Health Care (UHC) in the country, we are reminded that autism is only one of the many examples of disabilities where health inequalities are rampant. Autism, while sometimes considered a part of intellectual and developmental disabilities (IDD), is in fact a developmental disability (DD) that may not necessarily encompass intellectual disability. However, while both conditions fall under the umbrella of IDD, they are distinct in their manifestations. Autism primarily affects communication, social interaction, and behavior, while intellectual disability primarily impacts cognitive abilities and intelligence. Recognizing these differences is crucial to provide personalized support and create inclusive environments that acknowledge and celebrate the unique strengths and challenges of individuals with autism and other developmental disabilities. Thus, we call for enhancing multi-sectoral programs that would improve the lives of people with both intellectual and developmental disabilities, especially in the rural and remote areas of the Philippines. In developing countries where resources are limited, community-based tools that highlight the need for local policies and context-driven solutions could be developed to shift simple community paradigms in favor of the improvement of their quality of life. Policies such as institutionalizing early diagnostic tools and increasing hiring opportunities for these individuals are crucial for establishing sustainable practices that can further support their continuum of care. In addition, more accessible telemedicine and tele-education modalities should be explored and integrated into local and primary-care settings.^6^ As a response to this call, a community-based project integrating global health and human-centered design was established under the guidance of ThinkWell Institute to address the gap in the care and support for Filipino children with IDD.

## Responding to the Call—The Tinatangi Project

In the Philippines, there have been various initiatives done and organizations founded to help empower and support the needs of people with IDD. One of these is the Special Olympics Pilipinas, an organization that aims to offer year-round sports training and Olympic-style competitions for children and adults with intellectual disabilities, enabling them to enhance physical fitness and foster meaningful connections with their families, fellow athletes, and the community.^7^ Autism Society Philippines (ASP) is a non-profit organization that also provides support to families and individuals living with autism. They offer a wide range of services, including regular family support groups, training sessions on various topics, early detection and intervention consultancy, free community-based therapy services.^8^ However, to the authors’ knowledge, there have been no other initiatives done at the level of the local government which focus specifically on children with IDD, their caretakers, and how to support them. This is the first local initiative done as a product of a global health fellowship which was supported by ThinkWell Institute, consisting of a group of global and local mentors, and provided with access to health systems, policy, and entrepreneurship resources.

### Origins

As a brainchild of a Samya Rose Stumo Memorial Fellowship for Global Health cohort last 2022 and co-author of this article, the Tinatangi Project was originally founded last August 2022 to address the needs of children in a local special education center in the province of Bulacan, Philippines, to eventually expand to neighboring communities. Meaning precious and most beloved in Filipino, the word “tinatangi” embodies the project’s advocacy to improve the care and support for marginalized Filipinos with intellectual and developmental disabilities, as well as their families. Another term in Filipino is “tinatanggi” which means “to refuse, deny, or scorn.” Sadly, this is what children with IDD face every day, especially in developing countries like the Philippines. They often struggle to access basic services like education, employment, and healthcare. By removing stigma, prejudices, and discrimination, the Tinatangi Project believes that we can transform “tinatanggi” into “tinatangi,” a Filipino word that means most beloved, unique, and one and only.

Through a collective effort involving advocates, non-profit organizations, the local government of Marilao, Bulacan, healthcare professionals, and other stakeholders, the project aims to empower children with disabilities, promoting inclusion and breaking down discrimination. The project currently caters to 100 to 150 kids at the Special Education (SPED) Center in Marilao but it aims to expand to other rural and remote areas in the Philippines. Overall, it aspires to provide community-based tools that will enhance progressive policies, early interventions, and context-driven practices to further support and empower people with IDD.

### Data Collection and Processing

Aiming to first study the needs of the community, the project conducted thematic analysis from focused group discussions, and utilized various human-centered design tools such as empathy and persona mapping. The Inspiration phase involved being immersed within the community. Various interviews were conducted (online and in-person) with various stakeholders. Community visits were also done, observing mostly in the classroom setting, and with some house visits. Lastly, to gain a deeper understanding of the realities of parents of children with IDD, a separate focused group discussion was conducted to 24 parents from the community (either the father or the mother), highlighting their stories, and experience with regard to taking care of their children. Specifically, the 24 parents were asked in their native language 2 questions (nonverbatim) to facilitate discussion: “what are the blessings that you have realized in looking after your child?” and “what are the challenges that you have encountered in taking care of your child?” With the help of a scribe, concepts that arose in the discussion were written in cards, which acted as codes. With the participants’ consent as well, the session was also video recorded for documentation.

In the Ideation phase, a simple thematic analysis of the focused group discussion was done. The cards were organized into categories and ultimately into themes. Ultimately, the analysis revealed 3 interconnected concerns namely, facility concerns regarding the SPED Center, parental concerns about their children, and concerns of having the need for affordable allied services. First, concerns were raised regarding the SPED center. There was a strong clamor for having safe and nurturing spaces, both in schools and nearby areas, to protect these children from bullying and ensure they have inclusive environments such as having a playground. Secondly, parents expressed concerns about their children’s future, highlighting the need for financial and physical support for their children. The anxiety stems from the idea that they should always be available to care for their children’s activities of daily living. Lastly, there are significant challenges in accessing services like therapy, assessments, educational materials, and well-trained teachers. Similar to the rest of the country, especially during the pandemic, there was a need for flexible transitions to be able to provide services that are accessible and affordable to families with children with IDD.^9^

Another major point that also stood out in the discussion is the duality of the word *tinatangi*—that the parents know that their children are a little bit different, but they also deserve to be loved. Using these research tools, the Tinatangi Project was able to determine the importance of empowering the adults surrounding these children, and the community itself, to eventually make deep-seated change.

### Implementation

A common theme is apparent—importance of family and their immediate community in the lives of Filipino children with IDD. As such, various projects were started to address these concerns and raise awareness on the situation, stories, and potential of our Filipino children with IDD. First, in April 2023, Tinatangi held a session where children with IDD were encouraged to draw and paint freely. Through a partnership with a resin art maker based in Metro Manila, the artworks were photographed and sent to be made as customized designs. Their drawings were incorporated into resin art pieces (coasters, stickers, and phone accessories) that were successfully sold and fundraised for the community. A portion of the proceeds was used to create a play space, equipped with a balance beam for gross motor development, sensory pits for tactile and visual stimulation, art materials, and musical instruments. Termed as an “Advocacy Through Art fundraiser,” it was able to raise awareness within and outside of the community on the importance of both providing a therapeutic creative space and celebrating the worth and individuality of children with IDD. This partnership also extends beyond fundraising and becomes a catalyst that can improve the quality of lives of children with IDD through empowerment and social inclusion. Through this, the children are empowered in seeing their artworks be transformed into resin pieces that others cherish, and even provide funds for the play space that they and their peers and friends in the SPED Center will enjoy. They are also recognized for this accomplishment. This not only validates their artistic capabilities but also gives them a sense of accomplishment and belongingness. Second, in August 2023, Tinatangi also crafted an instructional and interactive primer (the Tinatangi Primer), that aims to educate and empower caretakers surrounding children with IDD. The primer included topics covering the definition of IDDs, politically correct and kinder terms for people with intellectual disabilities in the Philippines, and a directory of service delivery programs and facilities around the area. It also provided interactive worksheets that both parent and child can use, to provide education and learning in a holistic aspect. Lastly, various social media platforms were launched, sharing stories of various families and children with IDD in the community. Ultimately, starting with these, the Tinatangi Project envisions a society where individuals are seen and celebrated beyond their disabilities, and where everyone has access to quality healthcare and support, regardless of their background or circumstances.

## Conclusion

The pandemic has not only caused a global health crisis but has also highlighted the existing inequalities in our society, especially in terms of access to basic health care and services. For children with IDD, these challenges are amplified, not only for them but for their caretakers as well, making it even more important for us to support them. Inclusion should not just be a goal, but a fundamental value that guides us toward creating a more equitable and compassionate society where nobody is left behind. The Tinatangi Project serves as a beacon of hope for Filipino children with IDD since it seeks to promote inclusion and provide innovative and sustainable approaches to improve their quality of life. By working together and advancing multiple opportunities for children with IDD, we can create a more holistic community that benefits everyone.
